# Development and Evaluation of Bacteriophage Cocktail to Eradicate Biofilms Formed by an Extensively Drug-Resistant (XDR) *Pseudomonas aeruginosa*

**DOI:** 10.3390/v15020427

**Published:** 2023-02-02

**Authors:** Medhavi Vashisth, Anu Bala Jaglan, Shikha Yashveer, Priya Sharma, Priyanka Bardajatya, Nitin Virmani, Bidhan Chand Bera, Rajesh Kumar Vaid, Taruna Anand

**Affiliations:** 1 ICAR-National Research Centre on Equines, Hisar 125001, India; 2 Department of Molecular Biology and Biotechnology, College of Biotechnology, Chaudhary Charan Singh Haryana Agricultural University, Hisar 125004, India; 3 Department of Zoology and Aquaculture, College of Basic Sciences and Humanities, Chaudhary Charan Singh Haryana Agricultural University, Hisar 125004, India

**Keywords:** bacteriophage, *Pseudomonas aeruginosa*, extensively drug resistant (XDR), biofilm, phage cocktail

## Abstract

Extensive and multiple drug resistance in *P. aeruginosa* combined with the formation of biofilms is responsible for its high persistence in nosocomial infections. A sequential method to devise a suitable phage cocktail with a broad host range and high lytic efficiency against a biofilm forming XDR *P. aeruginosa* strain is presented here. Out of a total thirteen phages isolated against *P. aeruginosa*, five were selected on the basis of their high lytic spectra assessed using spot assay and productivity by efficiency of plating assay. Phages, after selection, were tested individually and in combinations of two-, three-, four-, and five-phage cocktails using liquid infection model. Out of total 22 combinations tested, the cocktail comprising four phages viz. φPA170, φPA172, φPA177, and φPA180 significantly inhibited the bacterial growth in liquid infection model (*p* < 0.0001). The minimal inhibitory dose of each phage in a cocktail was effectively reduced to >10 times than the individual dose in the inhibition of XDR *P. aeruginosa* host. Field emission-scanning electron microscopy was used to visualize phage cocktail mediated eradication of 4-day-old multi-layers of XDR *P. aeruginosa biofilms* from urinary catheters and glass cover slips, and was confirmed by absence of any viable cells. Differential bacterial inhibition was observed with different phage combinations where multiple phages were found to enhance the cocktail’s lytic range, but the addition of too many phages reduced the overall inhibition. This study elaborates an effective and sequential method for the preparation of a phage cocktail and evaluates its antimicrobial potential against biofilm forming XDR strains of *P. aeruginosa*.

## 1. Introduction

Extensive drug-resistant (XDR) *Pseudomonas aeruginosa*, in particular, is now recognized as one of the most prevalent causes of difficult-to-eradicate community-acquired and nosocomial infections [1,2,3]. Cystic fibrosis, pneumonia, malignant external otitis, skin and soft tissue infections, endocarditis, meningitis, and septicemia are some of the major infections caused by drug-resistant strains of *P. aeruginosa* [4,5]. Nosocomially, *P. aeruginosa* is associated in patients with cystic fibrosis, cancer, and burn cases. A vast number of innate and adapted mechanisms are adopted by *P. aeruginosa* to resist the bactericidal activity of antibiotics. Adherence with pilli and fimbrae leads to iron chelation from the host, thereby countering the host’s innate defenses [6]. The presence of outer membrane porins (OprD, OprF, OprJ, OprM, and OprN) imparts resistance against carbapenems, sulfonamides, fluoroquinolones, macrolides, and tetracycline [7]. Constitutively expressed β-lactamases and over-expression of efflux pumps in *P. aeruginosa* confer resistance to many antibiotics, thus leaving only a few end-line antibiotics such as colistin as treatment options. Formation of persistent biofilms, which are complex clusters of bacterial cells adhered to biotic or abiotic surfaces by extracellular polymeric substances (EPS), further complicates the treatment regimens devised for *P. aeruginosa* infections [8,9]. Biofilm is often considered a virulence factor [10]. The genetic loci *Alg*, *pel*, and *psl* encoded in *P. aeruginosa* genomes are responsible for the synthesis of EPS, which impedes the movement of antibiotics through the compactly packed layers of biofilm, thus rendering them ineffective. Biofilms also form a physical barrier that protects the inner cells from the outer environment and leads to the formation of persister cells, which are highly recalcitrant to the action of antibiotics due to their slower metabolic state [11,12,13]. The medical devices prone to biofilm formation include urinary catheters, cerebrospinal fluid shunts, endotracheal tubes, cardiac pacemakers, joint prostheses, contact lenses, and surgical instruments [14,15].

Recent years have witnessed a revived interest in phage therapy due to numerous advantages of bacteriophages, such as auto-dosing, less toxicity, lack of cross resistance, and their biofilm degradation ability attributed to exopolysaccharide depolymerases encoded in their genomes. These are the surface enzymes that penetrate and degrade bacterial EPS [16] and have been successfully utilized to treat severe infections caused by the formation of resistant biofilms of *P. aeruginosa* [17,18,19].

Bacteriophages target, infect, and lyse their host cells with high specificity. However, this high level of specificity severely limits the use of bacteriophage applications due to their inherent restricted host range [20,21]. While a limited host range is advantageous for phage typing, it is unfavorable for phage treatment regimens. In addition, the employment of a single phage type may stimulate the development of resistant mutants [22,23,24]. The most popular strategy for overcoming these constraints is the preparation of phage cocktails targeting a range of bacterial strains. Various studies have documented the development and use of phage cocktails for controlling bacterial growth in vitro [25,26,27], in animal models [28,29], and in humans [30,31]. Although there are a few available reports in the literature [32,33,34], in order to design a phage cocktail with near-certain therapeutic efficiency and with proven in vitro effects, a systematic design with proof of concept needs to be presented to explore the candidature of *P. aeruginosa* phages for inclusion in effective cocktails. Thus, in the current study, bacteriophages against various strains of *P. aeruginosa* were isolated and characterized. Different cocktails comprising suitable phage candidates with varied host range and lytic productivity were formulated and tested against an extensively drug-resistant (XDR) strain of *P. aeruginosa* for their bactericidal action in the liquid infection model and in the eradication of preformed biofilms to select the most effective cocktail of phages.

## 2. Materials and Methods

### 2.1. Bacterial Host Characterization

#### 2.1.1. Antibiotic Resistance Profiling Using Disk Diffusion Method

A total of 29 *Pseudomonas* strains were used to determine the antibiotic resistance/susceptibility against commonly used antibiotics following the CLSI guidelines [35]. Bacterial cultures having 0.5 McFarland standard equivalent growth were streaked on the Mueller Hinton agar plates, and antibiotic disks (HiMedia, Mumbai, India) were placed and incubated overnight at 37 °C. The diameter of the zone of clearance was measured and strains were classified as sensitive, MDR, or XDR on the basis of their resistance profiles according to the definitions provided by Magiorakos et al. (2012) [36]. The strains were categorized as MDR if they were non-susceptible to ≥1 agent in ≥3 antimicrobial categories and as XDR if they were non-susceptible to ≥1 agent in all but ≤2 categories [36].

#### 2.1.2. Assessment of Biofilm Forming Ability

*Pseudomonas* strains were assessed for their biofilm formation ability by the tube method [37] with a few modifications. Briefly, the overnight grown cultures of bacterial strains were inoculated in 2× LB medium supplemented with 1% glucose. After incubation for 96 h at 37 °C under static conditions, the planktonic cultures in the tubes were discarded. The adhered biofilms in the tubes were washed, fixed with methanol, and stained with 0.4% crystal violet for 15 min. Extra stain was drained, and the tubes were dried in air by inverting them. The presence of stained film lining the inner surface of the tube was indicative of the biofilm growth. The formation of a thin ring at the air-liquid interface was not considered positive for adherence. On the basis of the thickness of the stained film formed on the inner wall of the tubes, the bacterial strains were classified as strong biofilm-forming (very thick layer), moderate biofilm-forming (comparatively thinner layer), weak biofilm-forming (thin layer), and non-biofilm forming strains (no adherence). Exemplary images for different biofilm-forming strains are depicted with their respective results in Section 3.1.

### 2.2. Isolation and Propagation of Bacteriophages

Bacteriophages against *P. aeruginosa* were isolated from different sources from sewage water samples across Hisar city (29.170863, 75.716397), in Haryana, India, using the enrichment technique as described previously [38]. Briefly, 50 mL of sample was centrifuged to remove debris, and the resulting supernatant was incubated overnight at 37 °C with the targeted host bacteria (Fop416A, Fop426A, Fop489B, and Fop507C; refer to Supplementary Table S2). On the next day, the suspension was centrifuged and filtered through 0.22 μm PVDF syringe filter. The filtrate was spot tested on targeted bacterial lawn using the double layer agar (DLA) technique. The log phase bacterial culture (300 µL) was mixed with 3 mL of 0.7% soft agar in a molten state and plated on top of 1.5% nutrient agar. The filtrate forming a clear zone of lysis was used for plaque purification by picking and plating five times on the bacterial lawn using double layer agar assay (plaque assay).

For the preparation of bulk concentrates of bacteriophages, a method previously described by Anand et al. in 2018 was followed [38]. Briefly, a purified plaque was used to infect 5 mL of log phase bacterial culture re-suspended in 3 mL of SM buffer (NaCl—5.8 g/L; MgSO_4_—2.0 g/L; 1 M Tris (pH 7.5)—50 mL/L, sterile gelatin (2%)—5 mL/L). Bacteriophages were allowed to adsorb for 20 min at 4 °C under stationary conditions. The contents were transferred into fresh 50 mL nutrient media in a conical flask and incubated at 37 °C with vigorous shaking. After 18 h of incubation, the lysate was treated with chloroform and centrifuged to remove bacterial debris. NaCl was dissolved in the supernatant at a final concentration of 1 M and incubated on ice for 1 hr. After removal of debris by centrifugation, PEG8000 (Polyethylene Glycol 8000) (5% *w*/*v*) was added to the suspension and again incubated on ice overnight. Concentrated bacteriophage was obtained as a semi-transparent pellet and dissolved in SM buffer, and finally, pure bacteriophage suspension was extracted by chloroform treatment (1:1). The resulting suspension was serially diluted and plated using the DLA method to calculate the phage titre (PFU/mL).

### 2.3. Characterization of Bacteriophages

#### 2.3.1. Host Range and Cross Infectivity of Bacteriophages

The concentrated bacteriophage samples were diluted (1:10) in SM buffer and spotted (10 μL) on various *Pseudomonas* strains using DLA method. After overnight incubation at 37 °C, a clear zone of lysis indicated the lytic action of bacteriophage. On the basis of appearance of lysis zone, the lytic activity of the bacteriophages was characterized as highly/strongly lytic (clear zone of lysis on bacterial lawn), moderately lytic (clear zone with haziness), weakly lytic (lysis with substantial turbidity) and non-lytic (no lysis). The data of bacteriophage host range were used to generate a heat map using GraphPad Prism v.8.0.2 (263) (Dotmatics, San Diego, CA, USA).

#### 2.3.2. Lytic Productivity of Bacteriophages by Efficiency of Plating (EOP)

To determine the lytic productivity, bacteriophage samples were serially diluted. The successive dilutions ranging from 10^4^–10^10^ PFU/mL were spot plated (20 μL) using on bacterial lawn of all *Pseudomonas* strains separately and incubated overnight at 37 °C. The titres were calculated, and EOP was determined as follows:

$$EOP=\frac{Titre\;of\;phage\;on\;target\;bacterial\;strain}{Titre\;of\;phage\;on\;strain\;used\;for\;propagation}$$

Phage productivity in terms of EOP values was classified as described by Mirzaei and Nilsson, 2015 [39]:

High productivity          >0.5
Medium productivity     0.5–0.1
Low productivity        0.001–0.1
Inefficient productivity      <0.001

#### 2.3.3. Bacteriophage Structural Morphology Determination by Transmission Electron Microscopy (TEM)

For TEM, 10 μL of bacteriophage suspension (~1 × 10^10^ PFU/mL) in SM buffer was loaded onto a carbon-coated nickel grid and allowed to adsorb for 5 min, and the extra suspension was washed away with sterile distilled water. The grid was air dried for 5 min and negatively stained with 2% uranylacetete (pH 4). The samples were examined in a JEOL JEM-1011 transmission electron microscope (Jeol, Peabody, MA, USA) operating at 80 kV.

#### 2.3.4. Phage Stability Assay over a Range of Temperature and pH

For temperature sensitivity assessment, the bacteriophage suspensions were incubated individually at various temperature points: 4 °C, 25 °C, 37 °C, 45 °C, 55 °C, 65 °C, 70 °C, and 80 °C. For pH stability test, the phage suspension was mixed in a 1:10 ratio with pH buffers ranging from pH2, pH3, pH4, pH5, pH6, pH7, pH8, pH9 to pH10. After 1 h of incubation, each treatment was plated for titre determination using the PFU assay.

### 2.4. Microplate Single Phage or Cocktail Virulence Assay

#### 2.4.1. Determination of Optimum Multiplicity of Infection (MOI) of Bacteriophages

The phages exhibiting a broad spectrum of lysis and showing productive phage infection in terms of EOP against *Pseudomonas* strains were selected to formulate bacteriophage cocktails. Prior to testing the efficacy of cocktails, the individual phages were used to determine bacterial inactivation at different ratios. The optimal multiplicity of infection (MOI) was determined on the basis of the killing curves obtained for each phage at various MOIs ranging from 1 to 0.00001 against XDR *P. aeruginosa*. The 100 μL of bacterial culture (~1 × 10^7^ CFU/mL) in Mueller Hinton Broth No. 2 Control cations (CAMHB), (Himedia, Mumbai, India) was mixed with 100 μL of phage suspension in SM buffer at MOIs 1 (~1 × 10^7^ PFU/mL), MOI 0.1 (~1 × 10^6^ PFU/mL), MOI 0.01 (~1 × 10^5^ PFU/mL), MOI 0.001 (~1 × 10^4^ PFU/mL), MOI 0.0001 (~1 × 10^3^ PFU/mL), and MOI 0.00001(~1 × 10^2^ PFU/mL) separately in the wells of a flat-bottom 96-well microtest plate. The 100 μL of CAMHB + 100 μL of sterile SM buffer served as negative control, and 100 μL of bacterial culture + 100 μL of sterile SM buffer served as positive control. The plates were kept at 37 °C with shaking. The time-kill curves were determined successively for 10 h at every 15 min interval by measuring the OD_600nm_ in a Multiskan GO Microplate Spectrophotometer, using SkanIt™ Software (Thermo Scientific, Waltham, MA, USA, ver. 1.01.12).

#### 2.4.2. Formulation and Assessment of Bacteriophage Cocktails in Liquid Infection Assay

Twenty-two different cocktails were formulated by applying random combinations of bacteriophages with each other. The bacteriophages were mixed together in equal volumes to achieve the final concentration of less than the minimum inhibitory MOI for each phage. For each mix, bacteriophages were taken in concentrations 2X/3X/4X/5X respectively for 2-, 3-, 4-, or 5-phage cocktails so that when they were combined with other phages, the resulting final concentration of a phage in every cocktail remained the same, i.e., 1X. These cocktails were grouped in sets of two-, three-, four-, and all five bacteriophages together. A total of 100 μL of XDR *P. aeruginosa* strain (1 × 10^7^ CFU/mL) was added to the wells of a 96-well microtest plate. To these wells, different bacteriophage cocktails were added (100 μL) and incubated at 37 °C with shaking. The bacterial turbidity was measured as OD_600 nm_ at an interval of 15 min for 10 h in a Multiskan GO Microplate Spectrophotometer, using SkanIt™ Software (Thermo Scientific, ver. 1.01.12). The time-kill curves so obtained were compared for reduction in bacterial turbidity to evaluate the efficacy of the lytic activity of different cocktails.

### 2.5. Anti-Biofilm Assay of Bacteriophage Cocktail on Urinary Catheters and Borosilicate Glass

Biofilms of XDR *P. aeruginosa* strain were developed on the inner walls of urinary catheters and on borosilicate glass cover slips. For this, overnight-grown host bacterium in Luria Bertani (LB) broth was inoculated in the wells of a six-well tissue culture plate containing urinary catheters (1 × 0.5 cm sections) and borosilicate glass cover slips (18–22 mm) immersed in 2X LB broth supplemented with 1% glucose. The plate was incubated at 37 °C for 96 h and after every 12 h interval, and the wells were replenished with fresh sterile media for eight cycles (96 h). The catheters and cover slips were removed from the bacterial media and were re-immersed in the wells of a six-well tissue culture plate containing 6 mL of bacteriophage cocktail, and were incubated at 37 °C for 12 h with gentle shaking (30 rpm). A treatment group with sterile SM buffer was considered as positive control. Afterwards, the catheters and coverslips were removed from the wells, washed in PBS, fixed in 2.5% glutaraldehyde solution for 1 h at 4 °C and dehydrated using ethanol gradient process [25%, 50%, 75%, 90%, 100% (2X)]. Dehydrated catheters and cover slips were mounted on a copper stub, sputter coated with gold for 1 min, and visualized using Field-Emission Scanning Electron Microscopy (FE-SEM) in a JSM-7610F Plus Scanning electron microscope, Jeol, Akishima, Japan. Residual viable cell count was performed in both treated and non-treated groups using colony forming unit (CFU/mL) assay.

### 2.6. Statistical Analysis

Statistical analysis was performed in GraphPad Prism v.8.0.2 (263). For optimal MOI determination and bacteriophage cocktail action assay, the data were analyzed using two-way ANOVA with Tukey’s multiple comparisons test. A *p*-value < 0.01 was considered to be the threshold for significance. All the experiments were performed in triplicates. The mean values of replicates are depicted with the standard error of the mean.

## 3. Results

### 3.1. Host Bacterial Characterization and Selection of Biofilm-Forming XDR P. aeruginosa

Out of the 29 strains of *Pseudomonas*, 20 were categorized as MDR, and 1 was categorized as an XDR strain (VTCCBAA1047) (Supplementary Table S1). When tested for their biofilm-formation abilities over a period of 96 h using the tube method with crystal violet staining, out of 29 strains, 5 were found to be non-biofilm forming, 2 were weak biofilm forming, 4 were moderate biofilm forming, and 18 strains were found to be strong biofilm formers (Table 1 and Supplementary Figure S1). The *P. aeruginosa* strain (VTCCBAA1047) showed an extensive drug-resistance profile (XDR strain), and strong biofilm formation ability was selected to test the activity of the bacteriophage cocktail.

### 3.2. Isolation and Characterization of Bacteriophages

**Figure 1:**
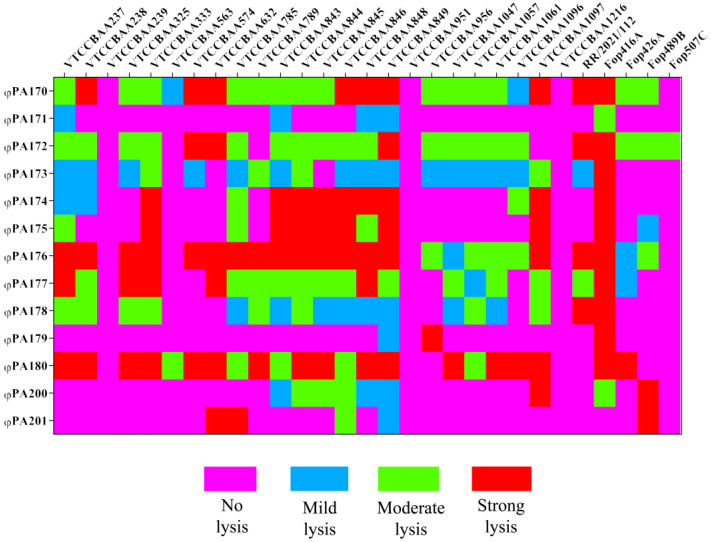
Heat map depicting host lytic spectrum of *P. aeruginosa* phages against various *Pseudomonas* strains.

A total of 13 bacteriophages were isolated using various strains of *Pseudomonas* described in Supplementary Table S1. These bacteriophages were accessioned as φPA170, φPA171, φPA172, φPA173, φPA174, φPA175, φPA176, φPA177, φPA178, φPA179, φPA180, φPA200, and φPA201 in Bacteriophage repository at National Centre for Veterinary Type Cultures, National Research Centre on Equines, Hisar, Haryana, India. These bacteriophages were tested for their lytic spectrum against various *Pseudomonas* strains using spot assay. On the basis of spot assay, four phages viz. φPA170 (86%), φPA176 (82%), φPA172 (79%), and φPA180 (75%) were found to have broad host range, while phages φPA179 (10%), φPA171 (17%), and φPA201 (17%) had a narrower host range (Figure 1). The phages φPA173 (68%), φPA174 (44%), φPA175 (41%), φPA177 (68%), φPA178 (62%), and φPA200 (31%) had comparatively moderate host range. Phage φPA170 lysed the maximum number of *Pseudomonas* strains (25/29) and was strongly lytic against 9 (number), moderately lytic against 14 (number), and mildly lytic against 2 (number) of strains. Similarly, φPA176 (24/29) and φPA180 (22/29) had slightly narrower host ranges, but both were strongly lytic on the 17 number, among these. To achieve a clearer understanding of lytic productivity of bacteriophages, EOP studies were also conducted. Only those phages which were capable of lysing the XDR *P. aeruginosa* VTCCBAA1047 strain were selected for EOP studies, which included φPA170, φPA172, φPA173, φPA176, φPA177, φPA178, and φPA180 phages. The results of EOP assay indicated that φPA180 had the highest productivity, followed by φPA176 on the 17 and 15 number of strains, respectively. Phage φPA180 also had the highest “High EOP/ lysed spot” ratio (0.77), followed by φPA176 (0.63) and φPA177 (0.35). However, amongst the seven phages tested for EOP assay, phage φPA176 and φPA178 did not yield a productive infection on XDR *P. aeruginosa* VTCCBAA1047, and hence were not further considered for cocktail design. Only φPA170, φPA172, φPA173, φPA177, and φPA180 phages, which were able to lyse XDR *P. aeruginosa*, were used for cocktail design. Comparison of results from spot assay and EOP assays are mentioned in Table 2. For individual EOP values and productivity results of phages, refer to Supplementary Table S2.

**Figure 2:**
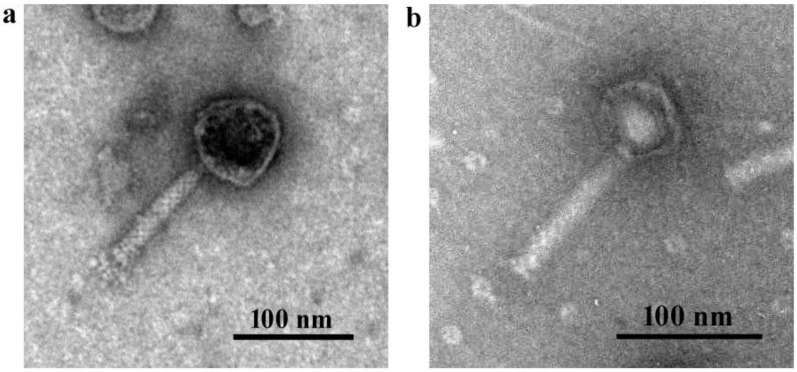
Transmission electron micrographs of (**a**) φPA170 and (**b**) φPA180. Both the phages belong to family *Myoviridae*, with a thick short tail, prominent base plate, tail pins, and tail fibres.

Considering the above results, φPA170 and φPA180 were selected on the basis of their high lytic spectrum and the productivity of the infection to form the cocktail against the XDR strain of *P. aeruginosa*. The structural morphology of phages φPA170 and φPA180 was studied using TEM. Morphological assessment showed that phages φPA170 and φPA180 belonged to family *Myoviridae*. Phage φPA170 exhibited an icosahedral head of 80.87 ± 1.81 nm diameter and a contractile tail 130.44 ± 6.36 nm in length in its non-contractile form and 48.93 ± 0.94 nm length in its contracted form with a thickness of 19.90 ± 0.65 nm. Prominent base plate and tail fibres are also visible in the micrograph. Phage φPA180 also had an icosahedral head of 56.18 ± 2.89 nm diameter, a contractile tail 119.85 ± 11.39 nm in length with 16.59 ± 1.65 nm width, and a thick base plate (Figure 2). Along with these two highly lytic myophages, three more phage isolates (φPA172, φPA173, and φPA177) that were able to lyse and infect XDR *P. aeruginosa* strain with efficient productivity (as demonstrated using EOP assay) were also included in the study in order to expand the lytic host range of phage cocktails.

**Figure 3:**
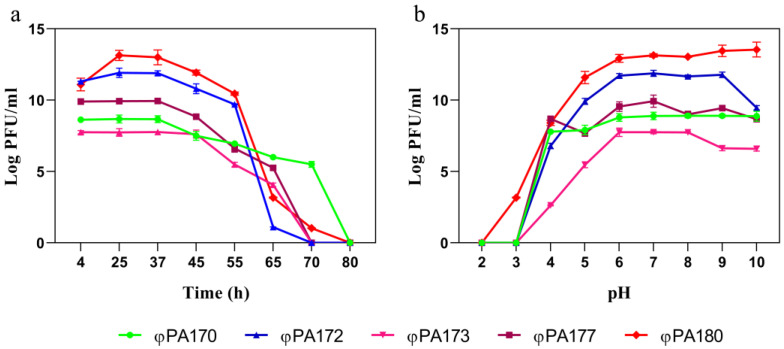
(**a**) Temperature sensitivity assay and (**b**) pH stability assay of phages φPA170, φPA172, φPA173, φPA177, and φPA180. Error bars depict standard deviation.

The sensitivity profile for a range of temperatures and pH of the selected phages are indicated in Figure 3. The phages were active over a different range of temperatures. Bacteriophages φPA172, φPA173, and φPA177 were inactivated at temperatures beyond 65 °C. Phages φPA170 and φPA180, though suffering considerable loss in titre, were able to form viable plaques at 70 °C. All the phages were inactivated during treatment at 80 °C for 1 hr. Phage φPA180 was able to produce plaques at acidic pH of 3 and was also stable at pH10. The rest of the phages were inactivated at pH value < 4. For most of the phages, a maximum stability was observed around pH 6–8. The structural proteome comparison of phages φPA170, φPA172, φPA173, φPA177, and φPA180 using SDS-PAGE is represented as Supplementary Figure S2 (included as a part of basic characterization details).

### 3.3. Microplate Single Phage or Cocktail Virulence Assay

**Figure 4:**
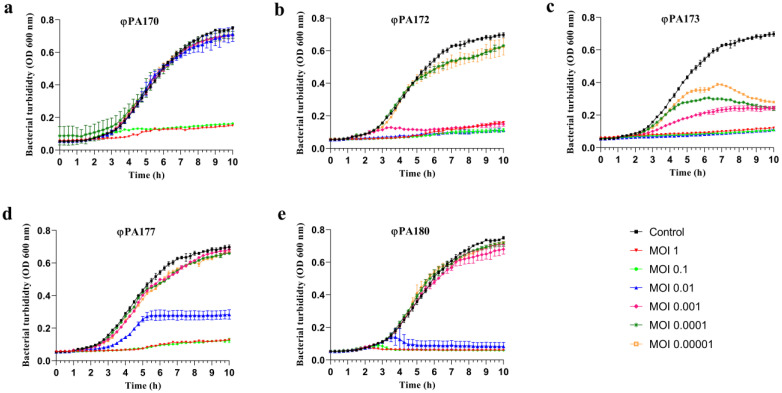
Inhibition of XDR *P. aeruginosa* VTCCBAA1047 growth by individual phages (**a**) φPA170, (**b**) φPA172, (**c**) φPA173, (**d**) φPA177, and (**e**) φPA180 in liquid infection models using time-kill assay over a time period of 10 h. Bacteriophages were used at MOIs ranging from 1 to 0.00001. Each point represents the mean of three replicates. The values of optical density used to plot graphs are blank (negative control) subtracted values. Each point represents the mean of three replicates. Error bars in the graph depict standard deviation from mean.

In order to harness the best lytic efficiency of phage cocktail, individual phages viz. φPA170, φPA172, φPA173, φPA177, and φPA180 were initially tested against XDR *P. aeruginosa* strain at varying MOIs viz. 1, 0.1, 0.01, 0.001, 0.0001, and 0.00001, and respective time-kill curves were obtained (Figure 4). Phage φPA170, φPA172, φPA173, φPA177, and φPA180 inhibited the bacterial growth up to MOIs 0.1, 0.001, 0.01, 0.1, and 0.01 respectively. Additionally, phages φPA173 and φPA177 limited the bacterial growth at MOIs 0.00001 and 0.01, respectively. On the basis of time-kill curve assay, phages φPA170, φPA172, φPA173, φPA177, and φPA180 were used at final concentrations of MOIs 0.01, 0.0001, 0.00001, 0.001, and 0.001, respectively, for synergy assessment in cocktail preparation.

**Figure 5:**
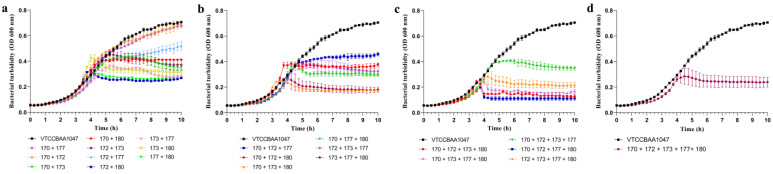
Lytic activity of bacteriophage cocktails against XDR *P. aeruginosa* VTCCBAA1047. A total twenty-two cocktails were designed with five phages in different combinations. Cocktail comprising (**a**) two phages, (**b**) three phages, (**c**) four phages, and (**d**) five phages were used to study inhibition of bacteria growth in liquid infection model using time-kill assay. The change in bacterial turbidity was measured at a 15 min interval successively for 10 h. All of the phages were used at a less-than-minimum inhibitory MOI. The values of optical density used to plot graphs are blank (negative control) subtracted values. Each point represents the mean of three replicates. Error bars in the graph depict standard deviation from mean.

Using different combinations of these five phages, a total of twenty-two cocktails were tested (Figure 5). Between the two-phage cocktails, the one comprising φPA172 + φPA180 was most effective followed by φ177 + φ180 in inhibition of the bacterial growth of XDR *P. aeruginosa*. Amongst the three-phage cocktails, φ170 + φ173 + φ180 was most effective followed by φ173 + φ177 + φ180 in inhibition of the bacterial growth of XDR *P. aeruginosa*, and amongst the four-phage cocktails, the one comprising φ170 + φ172 + φ177 + φ180 was most effective followed by φ170 + φ172 + φ173 + φ180 in inhibition of the bacterial growth of XDR *P. aeruginosa*. It is evident from the graph that the inhibition of the bacterial growth was best with four-phage cocktails and least with two-phage cocktails. The cocktail comprising φPA170 + φPA172 + φPA177 + φPA180 was able to completely inhibit the growth of XDR *P. aeruginosa*. Additionally, the cocktail comprising all five bacteriophages performed weaker than many of the four phage cocktails. We observed that the phage cocktails comprising φPA180 were the best in two-, three-, and four-phage cocktail groups to inhibit the bacterial growth.

### 3.4. Anti-Biofilm Assay of Bacteriophage Cocktail on Urinary Catheters and Borosilicate Glass

**Figure 6:**
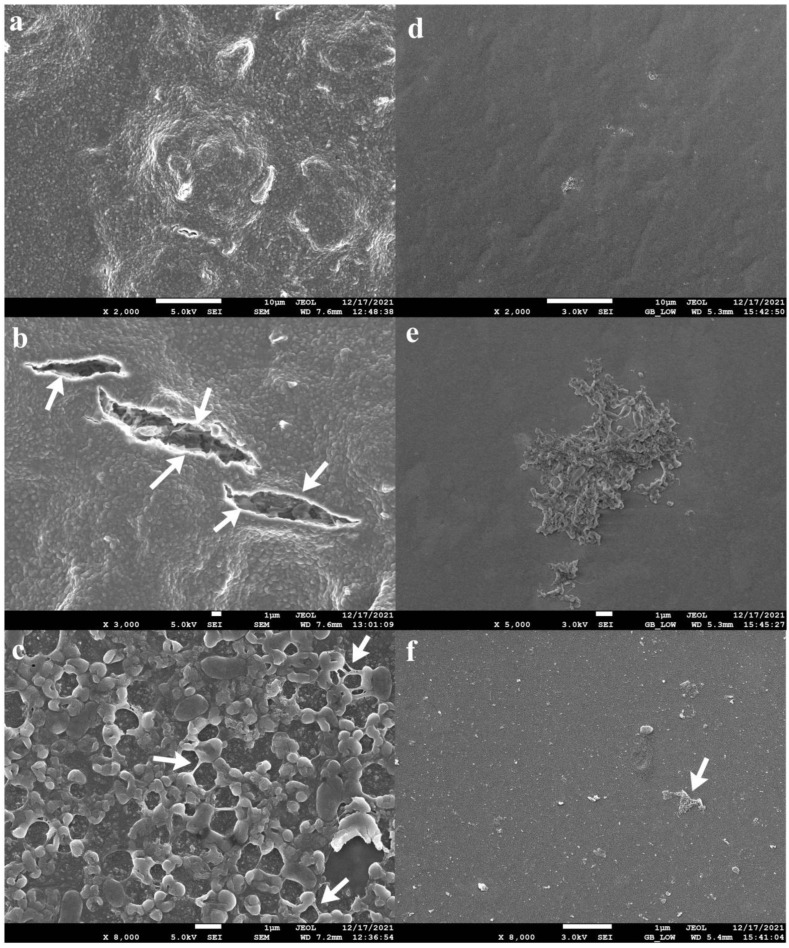
Biofilm eradication ability of phage cocktail comprising φPA170, φPA172, φPA173, and φPA180. Panels (**a**–**c**) represent 4-day-old biofilms developed on the inner walls of urinary catheter. Multilayered protrusions of tightly packed biofilm cells (**a**), and gelatinous matrix of EPS surrounding the cells (**c**) are observed. Arrows in (**b**,**c**) indicate cracks in biofilm multilayer and cell to cell EPS junctions respectively. Panels (**d**–**f**) represent eradicated biofilms after treatment with bacteriophage cocktail for 12 h. Arrows in (**f**) indicate cell debris. Biofilms were visualized in a JSM-7610F Plus Scanning electron microscope, Jeol, Akishima, Japan, after glutaraldehyde fixation and ethanol gradient dehydration.

**Figure 7:**
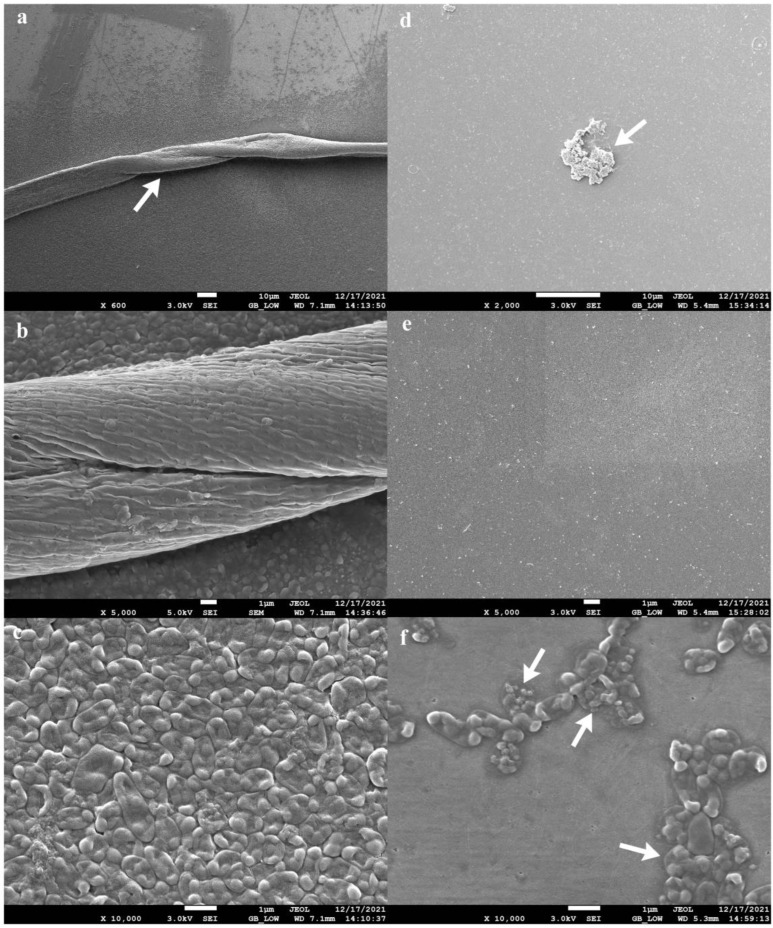
Biofilm eradication ability of phage cocktail comprising φPA170, φPA172, φPA173, and φPA180. Panels (**a**–**c**) represent 4-day-old biofilms developed on the borosilicate glass cover slips. Swivelled cords as indicated by arrows (**a**,**b**) and monolayers (**c**) of compactly adhered *P. aeruginosa* cells can be observed. Panels (**d**–**f**) represent eradicated biofilms and burst cell debris (indicated by arrows) after treatment with bacteriophage cocktail for 12 h. Biofilms were visualized in a JSM-7610F Plus Scanning electron microscope, Jeol, Akishima, Japan, after glutaraldehyde fixation and ethanol gradient dehydration.

The four-phage cocktail (φPA170 + φPA172 + φPA177 + φPA180), which resulted in the strongest inhibition of XDR *P. aeruginosa* strain was tested for its biofilm eradication ability. The 96-hour-old biofilms, developed on urinary catheters and glass cover slips, were used to demonstrate phage cocktail-mediated eradication. The biofilms developed on the inner walls of urinary catheter are indicated with prominent features such as multilayered protrusions of varying height and density arising from underlying layers of cells (Figure 6a). The core of the biofilm was also visible through breaks in the biofilm structure (Figure 6b). Cells adhering together and to the surface of the catheter in the gelatinous matrix of EPS could be seen (Figure 6c). After treatment with the bacteriophage cocktail, eradication of the biofilm (Figure 6d–f) was clearly noticeable without the presence of any intact cellular structure, and it was further confirmed by viable cell count using CFU assay. On cover slips, swivelled cords of *P. aeruginosa* cells compactly adhered together, and could be seen running through the biofilm structure (Figure 7a,b). Tightly packed cells of *P. aeruginosa* in monolayers could also be appreciated (Figure 7c). After the treatment, the surface of cover slips with eradicated biofilm (Figure 7d,e) and burst cell debris (Figure 7f) were visible.

## 4. Discussion

The worldwide spread of high-risk clones of XDR or MDR *P. aeruginosa* has become a serious public health threat [40,41,42,43,44]. The involvement of *P. aeruginosa* in biofilm-related infections on living tissues such as lung mucosa, cardiac valves, chronic wounds, sinusitis, dead tissues such as sequestra of bones [45,46], and on abiotic surfaces such as medical implants and surgical instruments [47,48] leads to the failure of established treatment regimens. With the scarcity of new drug molecules, researchers and clinicians worldwide are searching for effective alternatives to treat resistant bacterial infections [49]. Recently, bacteriophages have regained attention as potential antibacterial agents for curing difficult-to-treat infections. Several reports have demonstrated their therapeutic outcomes using in vitro studies [50,51,52], animal models [53,54,55,56,57], and human cases [58,59,60]. In the present investigation, the evaluation of individual and combined lytic action of bacteriophages against an XDR biofilm-forming *P. aeruginosa* strain in liquid infection assay as well as in eradication of biofilms was performed. A total of 13 bacteriophages were isolated and tested regarding their range of lytic action against a variety of *P. aeruginosa* strains classified as XDR/MDR/sensitive for a range of antibiotics and exhibiting varied biofilm-forming abilities.

Phage host range is a crucial character to consider for employing a particular phage in therapy. Spot assay, which is generally performed on double layer agar containing bacterial lawn, is used to determine its lysis capability on different bacterial strains. The bacteriophages used in this study had a varied spectrum of lysis against the tested *Pseudomonas* strains. Phage φPA170 followed by φPA176 and φPA180 lysed the maximum number of strains in spot assay. However, phage selection cannot be predicted solely based on the spot assay, which represents a bactericidal effect but does not completely indicate the true phage productivity. Spot assays have been reported to yield false positives due to the presence of bateriocins in the phage lysate [39]. Other probable mechanisms which might lead to a false positive in case of spot assay are cell lysis by abortive infections and phage-mediated lysis from without; in both these cases, clear plaques are formed without the formation of progeny virions [61]. A superior approach, however, is determining the EOP of bacteriophages while selecting them for therapy. In EOP assay, phage lysate is serially diluted and tested for formation of viable plaques, which provides a more substantiated proof of successful lytic infection. Thus, in the current study, the lytic potential of bacteriophages was further assessed by EOP assay. The results from EOP assay were observed to differ from those of spot assay. In the spot test, φPA170 lysed the maximum number of strains (86%); however, it showed three times lower productivity (High EOP/lysed spot ratio = 0.28) in EOP assay (Table 2). Phages φPA176 and φPA172 also performed comparatively lower in EOP assay than the spot assay, whereas results of φPA180 remained almost consistent for both EOP assay (High EOP/lysed spot ratio = 0.77) and spot assay (75%). Overall, φPA170 gave the best results in spot assay, and φPA180 gave the best results in EOP assay. Both of these bacteriophages belonged to family *Myoviridae*. Out of 223 *Pseudomonas* phages annotated with putative lytic proteins in UniProt database, 39% belonged to family *Myoviridae* of order caudovirales [62]. The bacteriophages from family *Myoviridae* are also linked with high bacterial lytic efficiency [63,64], and are also major component of bacteriophage cocktails [33,65,66]. Phages with broader host range are more suitable for therapy as they have more chances of infecting a large number of emerging strains of pathogenic bacteria, whereas propagation, storage, and clinical development of lytic phages with narrow host range become prohibitively costly as they require trials of many individual phages. So, the study of EOP of a bacteriophage is considered the most important determinant with respect to therapy and for indicating its lytic/lysogenic behaviour, as a lytic phage is reported to consistently yield clear plaques in a range of bacterial strains [67].

Bacteriophages are very specific in their action and this specificity can be a limiting factor when selecting a specific phage for therapy [68]. There are two ways to overcome this limitation: one is the identification of polyvalent phages [69] and the other is to formulate a bacteriophage cocktail with constituent phages having different host range and thus enabling lysis of a large number of strains [70]. Thus, keeping in mind the above-described results, we primarily selected two phages, i.e., φPA170 and φPA180, out of a total of thirteen phages to formulate a bacteriophage cocktail against XDR *P. aeruginosa*. To further increase the host range of the cocktail, φPA172, φPA173, and φPA177 were also selected as they had varying host ranges and were also able to produce plaques on the selected XDR *P. aeruginosa* host. All of the five bacteriophages were tested individually and in different combinations for their bactericidal activity against the targeted host. Individually, the phages were employed in the liquid infection assay against XDR *P. aeruginosa* at varying MOIs ranging from 0.1 to 0.00001. Assessment of MOI is an important factor when bacteriophages are used to control the bacterial load [71]. Bacteriophages in this study behaved in a varying manner over a range of MOIs due to differences in their lytic efficiencies. Phage φPA170 was able to inhibit bacterial growth only at higher MOIs of 1 and 0.1, whereas φPA173 lowered the bacterial density even at the lowest MOI used, i.e., 0.00001. Phages φPA172, φPA173 and φPA180 limited the bacterial growth at MOIs 0.001, 0.01, and 0.01 respectively. In all the phages except φPA180, there was a slight increment in bacterial OD after 10 h, which is an indicator of emergence of resistance [72]. However, for φPA180 at MOIs 1, 0.1, and 0.01, significant inhibition of bacterial growth up to 10 h was observed, indicating the probable potential of φPA180 for evading development of resistance. Surface modification of bacterial receptors has been suggested to be the most common resistance mechanism used by host cells to evade the attachment of bacteriophages [73], and bacteriophages incorporate modifications in their receptor binding proteins (RBPs) to overcome this resistance mediated by host cells. Phages with a broad host range may possess dual-receptor specificity or molecular mechanisms for diversification of RBPs, thus allowing them to switch between multiple hosts for receptor binding [69]. Bacteriophage φPA180, may be one such kind of phage, as depicted by its high EOP on 77% of tested bacterial strains and complete killing of bacterial cells without any observed resistance.

Further, when these phages were mixed and applied as cocktails in sets of two-, three-, four-, and five-phage suspensions, the highest bactericidal action was observed with the mixture of the four-phage cocktail (φPA170, φPA172, φPA177 and φPA180). Additionally, this four-phage cocktail was more effective for controlling the bacterial growth than any of the individual phage components. It is interesting to observe here that, overall, four-phage cocktails were better at bacterial killing than three-phage cocktails, which in turn were better than two-phage cocktails (Figure 5). The improved lytic efficiency may be based on the evolutionary approach, suggesting that diverse selection forces are more successful than individual ones in limiting the bacterial growth as well as in controlling the development of resistance [74]. Along with increasing the host range, multiple phages in a cocktail can also suppress the evolution of resistance, as the resistant mutant generated against one phage may be acted upon by another phage in the cocktail. However, sometimes the presence of too many types of phages together may also lead to competitive inhibition of bacteriophage action [75]. This was observed in our study where the cocktail comprising all five phages performed weaker than four-phage cocktail. One of the reasons for this phenomenon may be the targeting of the same receptor binding proteins (RBPs) of the host cell by more than one bacteriophage thus limiting/blocking the availability. Such competitive inhibition of phages has also been reported previously with phages infecting *Erwinina amylovora*, where the presence of one phage (ϕEa21-4) significantly lowered the replication rate of another phage (ϕEa35-70) in a cocktail [76]. Another report by Kim et al. (2020) also discusses the competition and loss of infectivity among phages in a cocktail if they target the same bacterial receptor [77].

The cocktail comprising φPA170, φPA172, φPA177, and φPA180 was also tested for its ability to eradicate biofilms from the inner walls of urinary catheter and borosilicate glass cover slips formed by XDR *P. aeruginosa*. Biofilms tolerate the action of antibiotics due to the physical barrier provided by the outermost layer of EPS, which contributes to enhanced resistance in biofilm communities by obstructing the movement of antibiotics to the inner layers of cells by directly binding to the drugs [78]. Bacteriophage-encoded polysaccharide depolymerases can degrade EPS [79], and many bacteriophages producing such enzymes have been discovered against Gram-negative bacteria [80]. The exopolysaccharide depolymerases of bacteriophages dissolve EPS, and then the persister cells residing in the innermost layers of biofilms become exposed to the bactericidal action of lytic bacteriophages or other antimicrobials [81]. The cocktail used in this study was able to successfully eradicate 4-day-old biofilms from both catheter walls and cover slips, and the degraded EPS without the presence of any intact cells can be seen under FE-SEM (8e, 9d, 9f).

The cocktail designed using the currently described methodology was effective in controlling the bacterial growth in liquid infection model and for biofilm eradication in vitro. Efficacy of various phage combinations was compared and analyzed, but how the concentration of individual phages in a cocktail affects its overall lytic efficiency has to be elucidated further. Studies involving safety and efficacy of phage cocktails in animal models and those exploring the role of phage adsorption kinetics with varying concentrations in cocktails are envisioned in order to provide a robust and reliable scientific framework for establishing protocols to employ phages in therapy.

## 5. Conclusions

This study presents a systematic method for the selection of phages to be incorporated in a phage cocktail to expand the host lytic range in order to achieve effective bactericidal action and lesser the propensity to generate resistance. Bacteriophages were selected after a thorough testing of their lytic profile using spot assay, EOP assay, and time-kill assay in a liquid infection model to ensure the formulation of an efficient phage cocktail superior to single phages in the eradication of XDR *P. aeruginosa* biofilms formed on medical devices such as urinary catheters. It was observed that different combinations of bacteriophages resulted in differential inhibition of bacterial growth. Increasing the targeted host strains by employing multiple phages can improve the lytic range of a cocktail; however, it was observed that the inclusion of too many phages may limit the efficacy of the phage cocktail due to competitive inhibition. So, evaluation of lytic bacteriophages to select the best combination becomes crucial before their application in therapy. Further studies comprising in vivo testing are envisaged to assess the efficacy and safety of bacteriophage cocktail against *P. aeruginosa* infections.

## Figures and Tables

**Table 1:** Biofilm formation ability of *Pseudomonas* strains (qualitative measurement by crystal violet staining using tube method).

Strength of Biofilm Formation	Number of Strains (Out of Total *n* = 29)	Percentage	Strains	Strength of Biofilm Formation as Visualized after CV Staining (Reference Images from Each Group)
Non-biofilm formers	5	17.24%	VTCCBAA238, VTCCBAA239, VTCCBAA333, VTCCBAA843, VTCCBAA1096	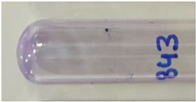
Weak biofilm formers	2	06.89%	VTCCBAA951, VTCCBAA1216	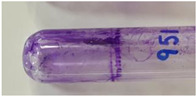
Moderate biofilm formers	4	13.79%	VTCCBAA574, VTCCBAA632, VTCCBAA785, VTCCBAA1057	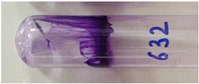
Strong biofilm formers	18	62.06%	VTCCBAA237, VTCCBAA325, VTCCBAA563, VTCCBAA789, VTCCBAA844, VTCCBAA845, VTCCBAA846, VTCCBAA848, VTCCBAA849, VTCCBAA956, VTCCBAA1061, VTCCBAA1047, VTCCBAA1097, RR/2021/112(571), Fop416A, Fop426A, Fop489B, Fop507C	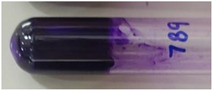

**Table 2:** Analysis of *P. aeruginosa* bacteriophages by comparison of their host lytic spectrum relative to efficiency of plating assay.

Bacteriophages	φPA 170	φPA 172	φPA 173	φPA 176	φPA 177	φPA 178	φPA 180
Total *Pseudomonas* strains used in the study (*n* = 29)							
Strains lysed in spot test ^@^	25	23	20	24	20	18	22
Percent lysed strains ^β^	86%	79%	68%	82%	68%	62%	75%
High EOP ^#^	7	4	1	15	7	2	17
Medium EOP ^$^	4	1	1	3	0	1	4
Low EOP ^*^	7	9	3	4	11	6	1
Inefficient plating ^λ^	7	9	15	2	2	9	0
Total EOP (=Total strains lysed)							
High EOP/Total EOP	0.28	0.17	0.05	0.63	0.35	0.11	0.77
High+Medium EOP/Total EOP	0.44	0.22	0.10	0.75	0.35	0.17	0.95

^@^ Calculated from Figure 1 using the *Pseudomonas* strains showing mild to strong lysis for each bacteriophage. ^β^ Calculated from total number of *Pseudomonas* strains (*n* = 29). ^#^ Based on number of *Pseudomonas* strains lysed with high EOP (Refer Supplementary Table S2. ^$^ Based on number of *Pseudomonas* strains lysed with medium EOP (Refer Supplementary Table S2). ^*^ Based on number of *Pseudomonas* strains lysed with low EOP (Refer Supplementary Table S2). ^λ^ Based on number of *Pseudomonas* strains with inefficient lysis in EOP assay (Refer Supplementary Table S2). Green and yellow cells represent respective maximum and minimum values in the rows.

## Data Availability

The bacterial strains and bacteriophages isolated and used during this study are deposited and available at Bacteriophage Repository, National Centre for Veterinary Type Cultures, ICAR-National Research Centre on Equines, Hisar, Haryana, India.

## Supplementary Materials

The following supporting information can be downloaded at: https://www.mdpi.com/article/10.3390/v15020427/s1, Table S1: Details of the bacterial strains used for bacteriophage isolation and characterization. Table S2: Efficiency of plating (EOP) of *P. aeruginosa* phages against different *Pseudomonas* strains. Table S3: Antibiogram of *Pseudomonas* strains. Figure S1: Biofilm formation ability of *Pseudomonas* strains. Figure S2: SDS-Polyacrylamide Gel Electrophoresis of phages viz. φPA170, φPA172, φPA173, φPA177, and φPA180.
