# Supplementary material for: Development and Evaluation of Bacteriophage Cocktail to Eradicate Biofilms Formed by an Extensively Drug-Resistant (XDR) Pseudomonas aeruginosa
Source: Viruses. 2023 Feb 2;15(2):427. doi: 10.3390/v15020427 (PMC9965693; doi:10.3390/v15020427)
Supplement: Supplementary file 1 [file viruses-15-00427-s001.zip › viruses-2162488-supplementary.pdf]

Title: Development and Evaluation of Bacteriophage Cocktail to Eradicate Biofilms Formed by an Extensively Drug-Resistant (XDR) *Pseudomonas aeruginosa*

Authors: Medhavi Vashisth [1,2], Anu Bala Jaglan [1,3], Shikha Yashveer [2], Priya Sharma [1], Priyanka Bardajatya [1], Nitin Virmani [1], Bidhan Chand Bera [1], Rajesh Kumar Vaid [1] and Taruna Anand [1,*]

Supplementary material for the article: The material has been provided in the order of appearance of the reference in the text.

Supplementary Table S1: Details of the bacterial strains used for bacteriophage isolation and characterization.

Sr. No.	NCVTC/ MTCC/ATCC -Accession no./ Lab ID	Bacterial strain	Source of isolation
1	VTCCBAA237	*Pseudomonas aeruginosa*	Gangrenous Mastitis
2	VTCCBAA238	*Pseudomonas aeruginosa*	Canine faecal Swab
3	VTCCBAA239	*Pseudomonas aeruginosa*	Hog Deer Nasal Swab
4	VTCCBAA325	*Pseudomonas aeruginosa*	Sheep Lung
5	VTCCBAA333	*Pseudomonas aeruginosa*	Camel
6	VTCCBAA563	*Pseudomonas aeruginosa*	Sheep
7	VTCCBAA574	*Pseudomonas aeruginosa*	Dog
8	VTCCBAA632	*Pseudomonas* species	Bovine cervical swab
9	VTCCBAA785	*Pseudomonas aeruginosa*	Sheep
10	VTCCBAA789	*Pseudomonas aeruginosa*	Canine pus from ear

11	VTCCBAA843	*Pseudomonas aeruginosa*	Turtle cloaca
12	VTCCBAA844	*Pseudomonas aeruginosa*	Mastitis
13	VTCCBAA845	*Pseudomonas aeruginosa*	Canine
14	VTCCBAA846	*Pseudomonas aeruginosa*	Human/Urine
15	VTCCBAA848	*Pseudomonas aeruginosa*	Canine gingival swab
16	VTCCBAA849	*Pseudomonas aeruginosa*	Canine
17	VTCCBAA951	*Pseudomonas* species	Sheep liver tissue
18	VTCCBAA956	*Pseudomonas aeruginosa*	Cattle milk from mastitis
19	VTCCBAA1047	*Pseudomonas aeruginosa*	Human/pus swab
20	VTCCBAA1057	*Pseudomonas aeruginosa*	Milk sample of bovine
21	VTCCBAA1061	*Pseudomonas aeruginosa*	Canine ear swab
22	VTCCBAA1096	*Pseudomonas aeruginosa*	Sheep Lung swab
23	VTCCBAA1097	*Pseudomonas aeruginosa*	Goat lung swab
24	VTCCBAA1216	*Pseudomonas aeruginosa*	Cattle faecal matter
25	RR/ 2021/ 112	*Pseudomonas aeruginosa*	Buffalo milk
26	Fop 416A	*Pseudomonas aeruginosa*	Community sewage
27	Fop 426A	*Pseudomonas aeruginosa*	Sewage water
28	Fop 489B	*Pseudomonas aeruginosa*	Biohazard waste, Dental ward
29	Fop 507C	*Pseudomonas aeruginosa*	Human urine

Supplementary Table S2: Efficiency of plating (EOP) of *P. aeruginosa* phages against different *Pseudomonas* strains.

**Bacteriophage**	**φPA170**		**φPA172**		**φPA173**		**φPA176**	
***Pseudomonas* strains (n=29)**	EOP value	Productivity	EOP value	Productivity	EOP value	Productivity	EOP value	Productivity
**VTCCBAA237**	0.007	Low	0.008	Low	0.000085	Inefficient	0.61	High
**VTCCBAA238**	0.06	Low	0.004	Low	0.000005	Inefficient	0.68	High
**VTCCBAA239**	-	-	-	-	-	-	-	-
**VTCCBAA325**	0.0002	Inefficient	0.0005	Inefficient	0	Inefficient	0.58	High
**VTCCBAA333**	0.0006	Inefficient	1E-07	Inefficient	0.0046	Low	0.81	High
**VTCCBAA563**	0.000002	Inefficient	-	-	-	-	-	-
**VTCCBAA574**	0.72	High	0.85	High	4.2E-06	Inefficient	0.51	High
**VTCCBAA632**	0.85	High	0.72	High	-	-	0.68	High
**VTCCBAA785**	0.12	Medium	0.00095	Inefficient	3.3E-06	Inefficient	0.85	High
**VTCCBAA789**	0.11	Medium	-	-	0.0062	Inefficient	0.27	Medium
**VTCCBAA843**	0.006	Low	0.00016	Inefficient	8.9E-07	Inefficient	0.81	High
**VTCCBAA844**	0.03	Low	0.00029	Inefficient	0.16	Medium	0.37	Medium
**VTCCBAA845**	0.00081	Inefficient	0.0107	Low	-	-	0.61	High
**VTCCBAA846**	0.25	Medium	0.029	Low	0.00125	Low	0.58	High
**VTCCBAA848**	0.93	High	0.0072	Low	1.42E-06	Inefficient	0.71	High
**VTCCBAA849**	0.56	High	0.64	High	2.5E-06	Inefficient	0.85	High
**VTCCBAA951**	-	-	-	-	-	-	-	
**VTCCBAA956**	0.015	Low	0.000079	Inefficient	0.000107	Inefficient	0.0126	Low
**VTCCBAA1047**	0.06	Low	0.0059	Low	0.00125	Low	0	Inefficient
**VTCCBAA1057**	0.00012	Inefficient	0.0057	Low	5.3E-06	Inefficient	0.0064	Low
**VTCCBAA1061**	0.15	Medium	0.0073	Low	0.000037	Inefficient	0.00133	Low
**VTCCBAA1096**	2.7E-06	Inefficient	0.0029	Low	2.6E-06	Inefficient	0.0014	Low

**VTCCBAA1097**	0.56	High	-	-	6E-07	Inefficient	0.98	High
**VTCCBAA1216**	-	-	-	-	-	-	-	-
**RR/2021/112**	0.81	High	0.33	Medium	1.6E-06	Inefficient	0.66	High
**Fop416A**	1	High	1	High	1	High	1	High
**Fop426A**	0.0003	Inefficient	0.00034	Inefficient	-	-	2.73E-05	Inefficient
**Fop489B**	0.089	Low	0.0002	Inefficient	-	-	0.109	Medium
**Fop507C**	-	-	0.00076	Inefficient	-	-	-	-

**Bacteriophage**	**φPA177**		**φPA178**		**φPA180**	
***Pseudomonas* strains (n=29)**	EOP value	Productivity	EOP value	Productivity	EOP value	Productivity
**VTCCBAA237**	0.74	High	0.0391	Low	0.78	High
**VTCCBAA238**	0.024	Low	0.026	Low	0.85	High
**VTCCBAA239**	-	-	-	-	-	-
**VTCCBAA325**	0.56	High	0.2	Medium	0.57	High
**VTCCBAA333**	0.7	High	0.02	Low	0.78	High
**VTCCBAA563**	-	-	-	-	0.17	Medium
**VTCCBAA574**	-	-	-	-	1.14	High
**VTCCBAA632**	0.81	High	-	-	0.85	High
**VTCCBAA785**	0.041	Low	3.91E-07	Inefficient	0.025	Low
**VTCCBAA789**	0.029	Low	0.023	Low	1.28	High
**VTCCBAA843**	0.017	Low	1.56E-08	Inefficient	0.42	Medium
**VTCCBAA844**	0.0046	Low	0.0126	Low	0.92	High
**VTCCBAA845**	0.5	High	2.17E-05	Inefficient	0.57	High
**VTCCBAA846**	0.065	Low	1.39E-07	Inefficient	0.135	Medium
**VTCCBAA848**	0.83	High	3E-07	Inefficient	0.57	High
**VTCCBAA849**	0.048	Low	9.1E-07	Inefficient	1	High
**VTCCBAA951**	-	-	-	-	-	-
**VTCCBAA956**	-	-	-	-	-	-

**VTCCBAA1047**	0.0058	Low	2.6E-07	Inefficient	0.78	High
**VTCCBAA1057**	0.00037	Inefficient	1.04E-05	Inefficient	0.185	Medium
**VTCCBAA1061**	0.069	Low	5.2E-10	Inefficient	0.607	High
**VTCCBAA1096**	-	-	-	-	0.71	High
**VTCCBAA1097**	0.081	Low	0.0173	Low	0.78	High
**VTCCBAA1216**	-	-	-	-	-	-
**RR/2021/112**	2	Low	0.78	High	-	-
**Fop416A**	1	High	1	High	0.85	High
**Fop426A**	8.1E-07	Inefficient	-	-	1	High
**Fop489B**	-	-	-	-	-	-
**Fop507C**	-	-	-	-	-	-

Interpretation for Phage productivity on the basis of EOP values:

High productivity > 0.5

Medium productivity 0.5 - 0.1

Low productivity 0.001 - 0.1

Inefficient productivity < 0.001

EOP values of bacteriophages on their respective original hosts are highlighted in yellow.

Blank cells represent absence of bacteriophage lytic activity

Supplementary Table S3. Antibiogram of *Pseudomonas* strains.

Antibiotic class		Penicillins	Monobactams	Cephalosporins				Cephalosporins + β lactamase inhibitors		Carbapenems		Polymyxins		Fluoroquinolones				Aminoglycosides			Macrolides
Strains. (n=29)	Status	PI 100	AT 30	CAZ 30	CPM 30	CTX 30	CEP 30	CAC 30/10	CEC 30/10	IPM 10	MRP 10	CL 10	PB 300	CIP 5	LE 5	NX 10	OF 5	AK 30	GEN 10	TOB 10	AZM 30
VTCCBAA237	MDR	S	S	S	S	R	R	I	S	S	S	S	S	S	S	S	S	S	S	S	R
VTCCBAA238	MDR	S	S	S	S	R	R	I	S	R	S	S	S	S	S	S	S	S	S	S	R
VTCCBAA239	Sensitive	S	S	S	S	S	S	S	S	R	S	S	S	S	S	S	S	S	S	S	S
VTCCBAA325	MDR	I	S	S	S	R	R	S	R	S	S	S	S	S	S	S	S	I	S	S	R
VTCCBAA333	MDR	R	I	R	R	R	R	R	R	S	S	S	S	S	S	S	S	I	S	S	R
VTCCBAA563	MDR	I	S	I	I	R	R	S	R	R	S	S	S	R	R	R	R	S	R	S	S
VTCCBAA574	MDR	S	I	S	S	R	R	S	R	R	R	S	S	S	R	I	R	S	S	S	R
VTCCBAA632*	Sensitive	S	S	S	S	S	R	S	S	S	S	S	S	S	S	S	S	S	S	S	R
VTCCBAA785	MDR	S	S	S	S	S	R	S	S	R	S	S	S	S	S	S	S	S	S	S	R
VTCCBAA789	MDR	S	R	I	I	R	R	I	R	R	I	S	S	S	R	R	R	S	S	S	R
VTCCBAA843	Sensitive	S	S	S	S	S	I	S	S	S	S	S	S	S	S	S	S	S	S	S	S

**VTCCBAA844**	**Sensitive**	S	S	S	S	S	R	S	S	S	S	S	S	S	S	S	S	S	S	S	S
**VTCCBAA845**	**Sensitive**	S	S	S	S	S	R	S	S	S	S	S	S	S	S	S	S	S	S	S	I
**VTCCBAA846**	**MDR**	S	I	S	R	R	R	S	R	S	S	S	S	S	S	S	S	S	S	S	I
**VTCCBAA848**	**Sensitive**	S	S	S	S	S	S	S	S	S	S	S	S	S	S	S	S	S	S	S	R
**VTCCBAA849**	**MDR**	S	S	S	S	S	R	S	S	R	S	S	S	S	S	S	S	S	S	S	R
**VTCCBAA951***	**MDR**	S	R	R	S	R	R	I	S	R	S	S	S	S	S	S	S	S	S	S	R
**VTCCBAA956**	**Sensitive**	S	S	S	S	S	R	S	S	S	S	S	S	S	S	S	S	S	S	S	I
**VTCCBAA1047**	**XDR**	I	I	S	S	R	R	I	I	R	R	S	S	S	R	R	R	S	R	S	R
**VTCCBAA1057**	**MDR**	S	S	S	R	R	R	I	R	R	S	S	S	S	S	S	S	S	S	S	R
**VTCCBAA1061**	**MDR**	S	S	S	R	R	R	S	R	R	R	S	S	S	S	S	S	S	S	S	I
**VTCCBAA1096**	**MDR**	S	S	S	I	R	R	S	S	S	S	R	S	S	S	S	S	S	S	S	R
**VTCCBAA1097**	**MDR**	I	S	S	S	R	I	S	S	S	S	S	S	S	S	S	S	S	S	S	I
**VTCCBAA1216**	**MDR**	S	I	S	S	S	R	S	S	S	S	S	S	S	S	S	S	S	S	S	I
**RR/ 2021/ 112**	**MDR**	S	I	S	R	I	R	S	S	R	S	S	S	R	R	R	R	R	R	R	R
**Fop416A**	**MDR**	I	I	S	I	I	R	S	I	R	S	S	S	S	S	S	S	S	S	S	R
**Fop 507C**	**MDR**	I	I	S	S	S	S	I	S	S	S	R	S	S	S	S	S	I	S	S	R
**Fop 426A**	**MDR**	I	R	S	R	R	R	I	R	R	R	S	S	I	R	R	R	S	S	S	I
**Fop 489B**	**MDR**	S	S	S	S	I	I	S	S	R	S	S	S	S	S	S	S	S	S	S	R

* indicates 2 *Pseudomonas* sp. and remaining 27 strains are *P. aeruginosa*; R - Resistant, I - Intermediately Resistant, S - Susceptible, MDR - Multiple drug resistant, XDR - Extensively drug resistant. Antibiotics are abbreviated as: PI100- Penicillin 100µg, AT30- Aztreonam 30µg, CAZ30- Ceftazidime 30µg, CPM30- Cefepime 30µg, CTX30- Cefotaxime 30µg, CEP30- Cephalothin 30µg, CAC 30/10- Ceftazidime-clavulanic acid 30/10µg, CEC30/10- Cefotaxime-clavulanic acid 30/10µg, IPM10- Imipenem 10µg, MRP10- Meropenem 10µg, CL10- Colistin 10µg, PB300- Polymyxin 300U, CIP5- Ciprofloxacin 5µg, LE5- Levofloxacin 5µg, NX10- Norfloxacin 10µg, OF5- Ofloxacin 5µg, AK30- Amikacin 30µg, GEN10- Gentamicin 10µg, TOB10- Tobramycin 10µg, AZM10- Azithromycin 10µg.

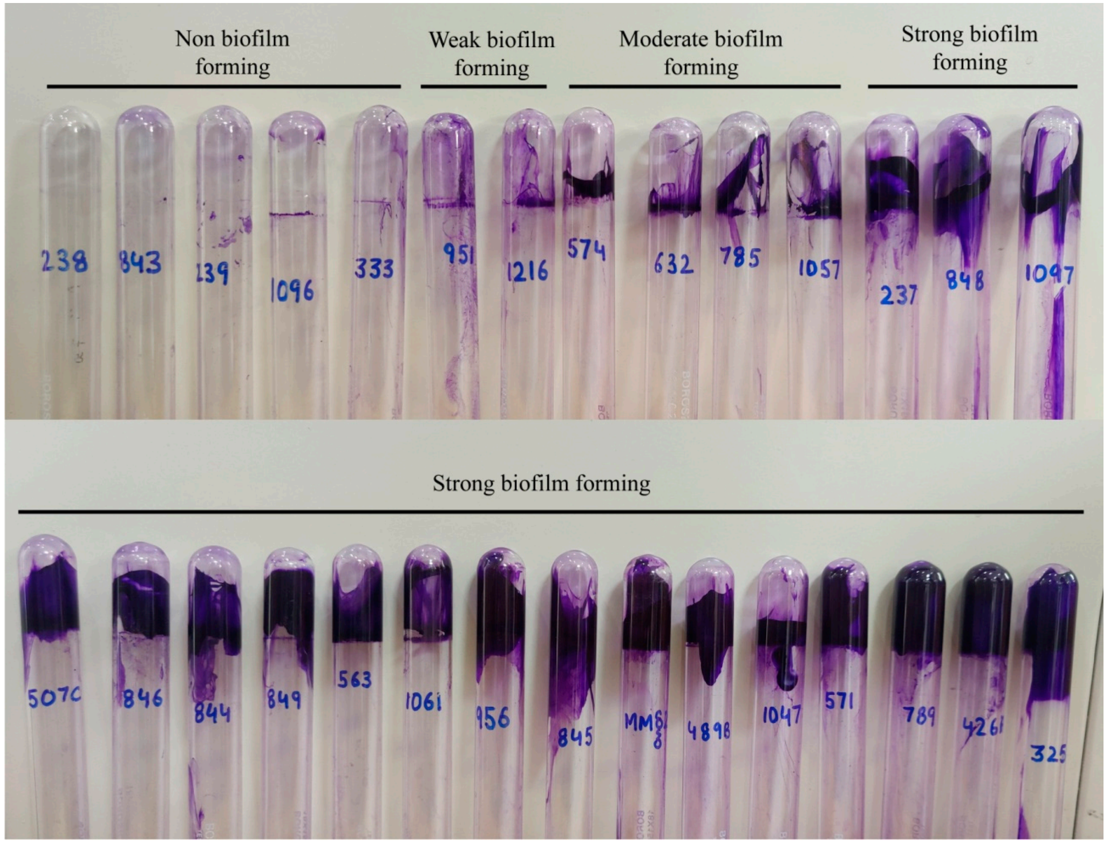

Supplementary Figure S1: Biofilm formation ability of *Pseudomonas* strains (qualitative measurement by crystal violet staining using tube method).

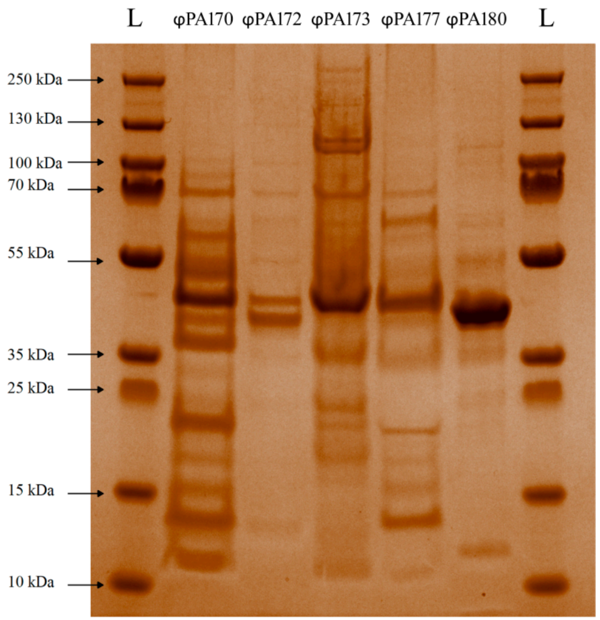

Supplementary Figure S2: SDS- Polyacrylamide Gel Electrophoresis of phages *viz.* φPA170, φPA172, φPA173, φPA177, and φPA180. The samples were prepared using manufacturer's protocol. Briefly, 20µl of the phage (1×10^{10} PFU/ml) was mixed with 15µl NuPAGE LDS Sample Buffer (4X), and 6µl of sample reducing agent and were heated for 15 minutes at 90°C in a dry bath. The samples were cooled to room temperature and were loaded into the wells of the NuPAGE 4-12% bis-tris gels and were electrophoresed at 80V for 3 hr till the bands of the protein marker were separated. The gel was then stained using Simply Blue Safe Stain (Invitrogen) for 1 hr on a gel rocker and was de-stained using distilled water and visualized in BioRad Gel Doc XR+ Gel Documentation System (BioRad, USA). Structural protein size was determined by comparing with PageRuler™ Plus Prestained Protein Ladder (26619), (Thermo Scientific) as standard using Image Lab software using (Bio Rad).
